# Upper Gastrointestinal Bleeding Secondary to Sodium Polystyrene Sulfonate Use: A Rare Adverse Effect of Commonly Prescribed Treatment

**DOI:** 10.1155/2024/6004323

**Published:** 2024-02-27

**Authors:** Hamzah Shariff, Shiva Naidoo, Ghazal Ghafari, Hongjie Li, Manisha Devi, Kishore Kumar

**Affiliations:** ^1^Geisinger Commonwealth School of Medicine, Scranton, Pennsylvania, USA; ^2^Department of Internal Medicine, Geisinger Wyoming Valley Medical Center, Wilkes Barre, Pennsylvania, USA; ^3^Department of Clinical and Laboratory Pathology, Geisinger Medical Center, Danville, Pennsylvania, USA; ^4^Department of Internal Medicine, Rosalind Franklin University of Medicine and Science, North Chicago, Illinois, USA; ^5^Department of Gastroenterology, Geisinger Community Medical Center, Scranton, Pennsylvania, USA

## Abstract

We report a case of a 62-year-old man who was brought in by emergency medical services after a fall and change in mental status. He was found to have severe hyperkalemia, acute kidney injury, and rhabdomyolysis. The hyperkalemia was treated with sodium polystyrene sulfonate (SPS). During hospitalization, he witnessed having black tarry stools along with a significant drop in hemoglobin. Endoscopic evaluation demonstrated nonbleeding large diffuse gastric ulcers with stigmata of recent bleeding, and ulcer biopsy revealed findings consistent with SPS-induced gastric ulceration. No other source of bleeding was localized, suggesting acute upper gastrointestinal bleeding due to SPS mucosal injury.

## 1. Introduction

Sodium polystyrene sulfonate (SPS) is a common comanagement in the treatment of hyperkalemia. It is well documented that the use of SPS is associated with gastrointestinal mucosal inflammation, leading to colonic mucosal injury and/or mucosal necrosis in severe cases. We present a case of acute upper gastrointestinal bleeding (UGIB), secondary to mucosal ulceration induced by SPS deposition confirmed on endoscopic evaluation and gastric ulcer histology.

## 2. Case Description

A 62-year-old man with a previous medical history of cerebrovascular accident, gastroesophageal reflux disease, hypertension, and chronic obstructive pulmonary disease was brought in via emergency medical services for evaluation of a fall at his home and acute change in mental status. In the emergency department, he was hypoxic and hemodynamically unstable. The laboratory workup was remarkable for acute kidney injury (AKI), hyperkalemia, and elevated creatine kinase (CK) level. Given inability to protect airways and presence of hypoxic respiratory failure, the patient was intubated and admitted to intensive care unit for aggressive resuscitation followed by nasogastric (NG) tube placement.

Additional laboratory abnormalities include high anion gap metabolic acidosis. The potassium level was reported as 8.8 mmol/L, serum creatinine of 12.5 mg/dL, and CK of >22,000 U/L. The initial hemoglobin level was 15.8 g/dL. Hyperkalemia was initially managed with dextrose, insulin, calcium gluconate, and SPS. The patient, however, was transitioned to renal replacement therapy given severe acidosis, oliguria, volume overload, and hyperkalemia.

**Figure 1:**
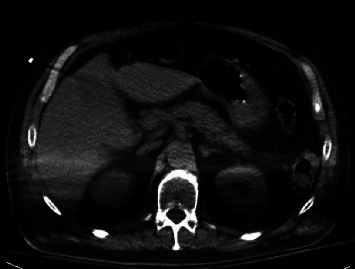
CT scan of the abdomen without evidence of gastric wall thickening or gas in the intramural layer.

During hospitalization, the patient developed ileus which was managed medically. On day 6 after admission, his hospital course was further complicated with coffee ground emesis and melena associated with a significantly downtrending hemoglobin level. A computed tomography (CT) scan of the abdomen was not significant for inflammatory changes or pneumatosis of the stomach (Figure 1).

**Figure 2:**
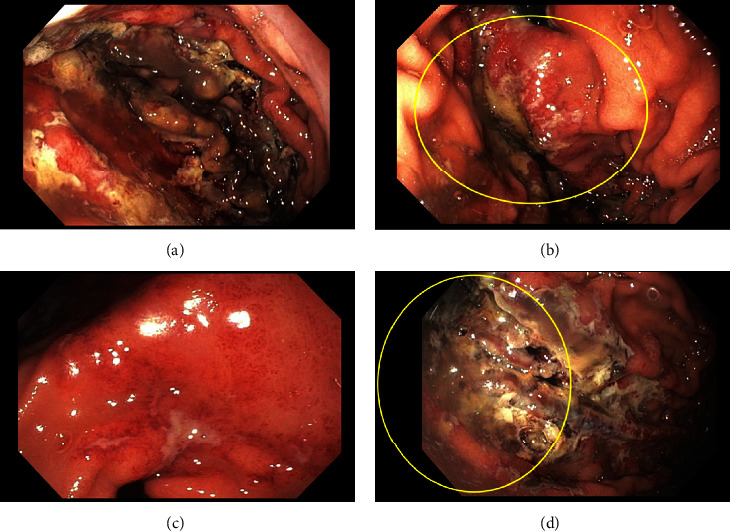
Endoscopic imaging of ulcerated gastric mucosa. (a) Mucosal ulceration of the gastric fundus, (b) ulceration and severe erythema of the gastric cardia on retroflexion view, (c) ulceration and severe erythema of the gastric prepyloric and pylorus, and (d) ulceration and erosion of the greater curvature of the gastric body.

**Figure 3:**
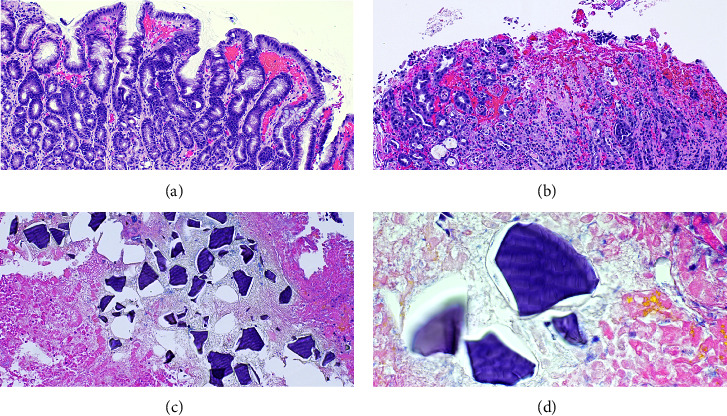
Gastric ulcer biopsy. (a) Reactive epithelium with corkscrew appearance of the surface epithelium (H & E, 100x). (b) Oxyntic mucosa with ulceration and marked reactive epithelial change (H & E, 100x). (c) Sodium polystyrene sulfonate crystals scattered in necrotic oxyntic mucosa (H & E, 100x). (d) Magnified image of sodium polystyrene sulfonate crystals with fish scale appearance (H & E, 400x).

On day 7, the patient then underwent esophagogastroduodenoscopy (EGD) which revealed severe esophagitis, nonbleeding large diffuse ulcerations involving the gastric fundus, body, antrum, and duodenum (Figure 2). Gastric ulcer biopsy results showed oxyntic mucosa with ulceration, marked reactive epithelial change, necrosis, and rare refractile but nonpolarizable crystals in a fish scale pattern suggestive of SPS-induced gastritis (Figure 3). The ulcers were then treated with proton pump inhibitor therapy. His hemoglobin remained stable, and the patient was discharged to a skilled nursing facility after three weeks of a complicated hospital course.

## 3. Discussion

Hyperkalemia is a common but critical electrolyte abnormality that requires close evaluation and immediate treatment. In addition to monitoring electrocardiogram changes and immediate cardiac membrane stabilization with intravenous calcium or insulin with dextrose, potassium binders are utilized for potassium elimination [1]. SPS, a resin that exchanges potassium for sodium in the colon, has been used for management of mild to moderate hyperkalemia for decades despite no reliable randomized clinical trials to determine efficacy and safety. Therefore, guidelines on usage are limited. A suggested approach is to administer SPS 15–60 g by mouth if loop diuretics are contraindicated due to end stage renal failure or severe hypovolemia [2]. The FDA in 2009 stated SPS is contraindicated in obstructive bowel disease and warned the risk of colonic injury including bleeding, ischemia, and perforation [3]. The majority of gastrointestinal adverse events was associated with concomitant use of sorbitol and is thus avoided with SPS administration.

A proposed mechanism of SPS on bowel injury, with the inclusion of sorbitol, can cause gastric injury as a cathartic agent. Also, SPS itself may cause injury through osmotic action and vasospasm of the intestinal vasculature [4]. Intestinal wall damage may even include the risk of mortality of 33% when diagnosis is established. SPS without sorbitol was less likely to have a statistically significant necrosis and ulceration association; however, no significant difference was found in preparations with sorbitol [4]. Biopsy findings of the ulceration typically show refractile, nonpolarizable crystals lightly basophilic on hematoxylin and eosin stain in a mosaic pattern [5]. Our patient was noted to have a large decrease in hemoglobin after administration of SPS to manage his hyperkalemia in the setting of hemodynamic instability and AKI. EGD findings included esophagitis and multiple nonbleeding diffuse gastric ulcerations of the stomach and duodenum. The biopsy results of the gastric ulcers confirmed the presence of refractile, nonpolarizable crystals and necrosis which correlated with SPS-induced gastritis. Given the clinical presentation, results of EGD and gastric ulcer biopsy, and no other source of bleeding, SPS was deemed the etiology of UGIB in this patient. This is one of the rare adverse effects of SPS therapy rather than the common finding of colonic injury with or without lower gastrointestinal bleeding [4, 6]. Ulceration of the antrum presenting as an UGIB due to SPS has been previously demonstrated, but diffuse involvement of the stomach resulting in UGIB as seen in our patient has not been reported [5]. Gastric ischemia may have been involved as the patient presented with hypotension which may have contributed to decreased perfusion of the stomach and increased susceptibility to damage by SPS [7]. Renal involvement was suspected to exacerbate SPS-induced gastrointestinal wall injury possibly related to angiotensin II-mediated splanchnic vasoconstriction [4, 8]. Development of an ileus may have slowed SPS transit and increased contact with the gastric and bowel mucosa. Gastric emphysema and emphysematous gastritis were considered given similar endoscopic findings and NG tube placement though they were ruled out as gastric pneumatosis was not demonstrated on the CT of the abdomen [9, 10].

The cautionary use of SPS should be implemented especially in patients with cellular hypoxia due to decreased blood pressure or systematic oxygen deprivation. Additionally, the adverse effect of SPS seems to be more frequent in patients with renal insufficiency [4]. Thus, other modalities for the management of hyperkalemia are prioritized. Early hemodialysis may prevent the increased risk of complications associated with SPS. However, this approach depends on vascular access and equipment availability and can be complicated by fatal arrhythmia due to rapid potassium clearance [11, 12]. Alternative novel potassium-lowering agents may be considered. Sodium zirconium cyclosilicate (SZC) and patiromer are well-tolerated oral potassium binders with higher affinities towards potassium [13, 14]. They have been recently approved by the National Institute for Health and Care Excellence guidelines to manage acute life-threatening hyperkalemia or persistent hyperkalemia while treating with renin-angiotensin-aldosterone system inhibitors in the setting of chronic kidney disease stages 3b to 5 or heart failure [15]. Clinical trials have demonstrated the achievement of normokalemia with both potassium binders in over 90% of subjects within 48 hours [16–18]. Patiromer has been shown to significantly decrease potassium levels over the course of 4 weeks and maintained normokalemia at 52 weeks, thus suggesting its long-term efficacy for the treatment of hyperkalemia [19, 20]. A phase II trial suggested the benefit of SZC use in acute hyperkalemia as it yielded a greater reduction in serum potassium with insulin and glucose compared to insulin and glucose alone within 2 hours of administration [21]. Although no serious gastrointestinal injuries have been reported, mild to moderate gastrointestinal upset is a common side effect associated with SZC and patiromer [16, 18–21]. In addition, given patiromer can bind magnesium and other drugs in a nonselective manner, monitoring for hypomagnesemia and ensuring at least a three-hour interval between the administration of patiromer and other oral medications are recommended [19, 20, 22, 23]. According to the 2021 American College of Cardiology Expert Consensus Decision Pathway and Kidney Disease: Improving Global Outcomes Clinical practice guidelines, these potassium binders are appropriate for achieving normokalemia, but more long-term data are necessary to establish the safety and efficacy of these drugs with certainty [24, 25].

## 4. Conclusion

The use of SPS is associated with gastrointestinal mucosal injury and may lead to diffuse gastric ulcerations, presenting as an acute UGIB. Further research is necessary to elucidate a clear mechanism of mucosal injury. Administration of SPS should be performed with caution while considering other potassium-lowering modalities when treating hyperkalemia to prevent gastrointestinal bleeding and clinical decompensation.

## Data Availability

All data underlying the results are available as part of the article, and no additional data are required.
